# Association of the single-point insulin sensitivity estimator with arterial stiffness: a cross-sectional analysis

**DOI:** 10.3389/fendo.2026.1858246

**Published:** 2026-05-18

**Authors:** Yuzhou Liu, Xinyue Guo, Zhe Meng, Xijia Wang, Hongrui Chen

**Affiliations:** 1 Department of Cardiology, The First Affiliated Hospital of Zhengzhou University, Zhengzhou, Henan, China; 2 Ophthalmological and Optometric Medicine, Beijing Tongren Hospital, Capital Medical University, Beijing, China

**Keywords:** arterial stiffness, brachial-ankle pulse wave velocity, insulin sensitivity, Japanese adults, single-point insulin sensitivity estimator

## Abstract

**Background:**

Arterial stiffness is an early marker of vascular damage and cardiovascular risk. SPISE is a simple insulin-free surrogate of insulin sensitivity, but its association with arterial stiffness remains unclear. This study assessed the association between SPISE and brachial-ankle pulse wave velocity (baPWV) in Japanese adults.

**Methods:**

A cross-sectional dataset of 912 adults from a health screening program in Gifu, Japan, was analyzed. SPISE was evaluated in two ways, including per-unit variation and quartile grouping. The independent association of SPISE with baPWV was estimated using linear regression techniques. To further explore the pattern of association, spline-based modeling was introduced, and subgroup and sensitivity analyses were further conducted to assess the robustness of the findings.

**Results:**

Higher SPISE levels were significantly associated with lower baPWV. With all covariates taken into account, baPWV decreased by 8.48 cm/s for each 1-unit higher SPISE (β = -8.48, 95% CI: -16.00 to -0.96; P = 0.027). Relative to the lowest quartile, the highest SPISE quartile had significantly lower baPWV (β = -56.17, 95% CI: -101.49 to -10.85; P = 0.015), with a significant trend across quartiles (P for trend = 0.013). The association was generally linear and consistent across subgroups. Sensitivity analyses showed that the association was robust to lipid adjustment but attenuated and changed direction after further adjustment for SBP, DBP, and fasting plasma glucose.

**Conclusion:**

Higher SPISE was generally associated with lower baPWV in Japanese adults and may serve as an easily obtainable metabolic marker associated with arterial stiffness.

## 1 Introduction

Arterial stiffness, a hallmark of vascular aging and early vascular injury, is recognized as a key pathophysiological process preceding the onset of cardiovascular events. It exhibits a pronounced age-related distribution in the general population. A large European study based on data from 13 centers across 8 countries demonstrated that pulse wave velocity (PWV) increases progressively with advancing age and higher blood pressure levels, highlighting the growing burden of arterial stiffness in the aging population (1). As a noninvasive index of arterial stiffness, PWV is also widely recognized as an indicator of cardiovascular risk and target organ damage (2). Among the available PWV-derived measures, brachial-ankle pulse wave velocity (baPWV) is particularly suitable for large-scale studies because it is simple, practical, and reproducible. In addition, baPWV has demonstrated value in detecting subclinical vascular injury at an early stage and refining cardiovascular risk assessment (3).

The development of arterial stiffness is influenced by multiple cardiometabolic risk factors, with insulin resistance considered an important pathway through which metabolic dysfunction contributes to vascular injury. Insulin resistance can impair the PI3K/Akt/eNOS signaling pathway, reduce nitric oxide bioavailability, and amplify oxidative stress and low-grade inflammation, thereby disrupting endothelial homeostasis (4). In turn, metabolic and vascular insulin resistance may synergistically aggravate abnormalities in vascular tone regulation, blood flow control, and arterial wall remodeling, ultimately accelerating the progression of arterial stiffening (5). Importantly, this relationship is not confined to overtly high-risk metabolic populations; consistent associations involving insulin resistance–related surrogate indices and measures of arterial stiffness, including PWV, baPWV, CAVI, and AIx, have also been observed in general and apparently healthy populations (6–8). Moreover, arterial stiffness may represent a critical intermediate pathway through which insulin resistance contributes to atherosclerotic cardiovascular disease (9), providing a strong rationale for evaluating early vascular injury from the perspective of insulin sensitivity.

Given the important role of insulin resistance in arterial stiffening, a simple and reliable indicator of insulin sensitivity may have considerable value for the early identification of vascular damage. Calculated from BMI, TG, and HDL-C, SPISE provides an indirect assessment of insulin sensitivity without the need for insulin measurement. Its ease of use, affordability, and feasibility make it attractive for population-based research and routine clinical screening (10). Because its components reflect both adiposity and insulin resistance-related lipid dysregulation, SPISE may indirectly capture the metabolic phenotype of impaired insulin sensitivity. Emerging evidence suggests that SPISE has potential utility in identifying insulin resistance and improving cardiometabolic risk stratification.

Existing studies on SPISE have mainly addressed metabolic syndrome, dysglycemia, fatty liver disease, and cardiovascular outcomes (10–14). In contrast, evidence regarding its relevance to arterial stiffness remains limited, particularly for peripheral or systemic arterial stiffness measured by baPWV. It also remains unclear whether any observed relationship can be explained by conventional cardiovascular risk factors or whether similar patterns are present in different population strata. Against this background, the association between SPISE and baPWV was examined, with additional stratified analyses performed to determine the robustness of the findings in different subgroups and to further clarify the potential value of SPISE for early detection of vascular injury.

## 2 Materials and methods

### 2.1 Study design and data source

Publicly available health check-up data were used for this retrospective cross-sectional analysis. These data were derived from adults who participated in routine health screening in Gifu, Japan, between March 2004 and December 2012, and were subsequently deposited in the Dryad repository for public access.

### 2.2 Ethical statement

The original study was approved by the relevant institutional ethics committee and was conducted in accordance with the Declaration of Helsinki (15). Written informed consent was obtained from all participants during the original investigation. The present study was a secondary analysis of de-identified, publicly available data; therefore, additional ethical approval was not required (16).

### 2.3 Study population

Participants eligible for the present analysis were adults from the original health check-up cohort who had available baPWV measurements. Participants with an ankle-brachial index (ABI) below 0.95 were not included in the original dataset, as a low ABI may compromise the accuracy of baPWV measurement. Pregnant women, women who tested positive for hepatitis B surface antigen or hepatitis C antibody, and those receiving oral contraceptives or hormone replacement therapy were excluded from the original dataset. After all exclusions, 912 participants remained in the study population (17).

### 2.4 Exposure and outcome assessment

#### 2.4.1 Baseline data and covariates

The dataset provided information on a range of baseline characteristics, including age, sex, BMI, systolic and diastolic blood pressure, liver enzyme levels (AST, ALT, and GGT), fasting plasma glucose, uric acid, lipid parameters (TC, TG, HDL-C, and LDL-C), eGFR, ABI, alcohol intake, smoking status, exercise habits, and fatty liver status. The adjustment of covariates is based on previous literature (8).

#### 2.4.2 Exposure and outcome variables

The main exposure variable was SPISE, an insulin-independent index calculated from BMI, TG, and HDL-C to reflect insulin sensitivity. SPISE was calculated as follows: SPISE = 600 × HDL-C^0.185/(TG^0.2 × BMI^1.338) (18, 19). SPISE was evaluated both as a continuous variable and as a categorical variable grouped by quartiles (Q1, ≤6.46; Q2, 6.46–7.84; Q3, 7.84–9.54; and Q4, ≥9.54).

The study outcome was baPWV, which was treated as a continuous marker of arterial stiffness. baPWV was measured using an automated arteriosclerosis detection device with participants in the supine position. Measurements were recorded on both sides at the same time, and the average of the two values was entered into the analysis.

### 2.5 Statistical analysis

Statistical analyses were performed using R version 4.3.1. Continuous variables are presented as mean ± SD or median (IQR), and categorical variables as numbers and percentages. Differences in baseline characteristics across SPISE quartiles were compared using the one-way ANOVA or Kruskal-Wallis test for continuous variables and the chi-square test or Fisher’s exact test for categorical variables.

Univariable linear regression was used as an initial step to evaluate the relationship of each candidate variable with baPWV. Variables of interest were then further assessed in multivariable linear regression models, and effect estimates are presented as regression coefficients (β) with corresponding 95% confidence intervals (CIs). SPISE was modeled as a continuous measure and by quartiles, with the first quartile taken as the reference category. To assess the linear trend across SPISE quartiles, the quartile groups were modeled as an ordinal variable.

Three regression models were fitted with stepwise adjustment. The basic model included age and sex. Liver enzyme markers, UA, eGFR, and ABI were then added, followed by alcohol consumption and smoking status in the final model. To further characterize the association between SPISE and baPWV, restricted cubic spline analysis was performed (20), and both the overall association and potential nonlinearity were examined (21). To evaluate the robustness of the results, sensitivity analyses were conducted with additional adjustment for TC and LDL-C, as well as for SBP, DBP, and fasting plasma glucose in separate models. To examine whether the association varied across populations, stratified analyses were conducted by age, sex, eGFR, alcohol consumption, smoking status, and exercise habits, and corresponding interaction terms were tested (22). Two-sided tests were used throughout, and results with P <0.05 were regarded as statistically significant.

## 3 Results

### 3.1 Characteristics of participants according to SPISE quartiles

The final analytic sample consisted of 912 participants, who were grouped into four quartiles based on SPISE levels. Their baseline characteristics are summarized in **Table 1**. Age was comparable across the four groups (P = 0.073), whereas the distribution of sex differed significantly among them (P < 0.001). Among the 912 participants, 592 were men and 320 were women. The proportion of men declined across increasing SPISE quartiles. baPWV also differed significantly among SPISE quartiles (P < 0.001), with lower values observed at higher SPISE levels.

**Table 1:** Baseline characteristic of the study population according to SPISE.

Variables	Total (n = 912)	Q1 (n = 228)	Q2 (n = 228)	Q3 (n = 228)	Q4 (n = 228)	P value
**baPWV**	1415.75 ± 246.25	1451.70 ± 232.10	1447.62 ± 261.84	1415.98 ± 266.91	1347.69 ± 206.68	***< 0.001***
**Sex, n (%)**						***< 0.001***
Male	592 (64.91)	194 (85.09)	187 (82.02)	125 (54.82)	86 (37.72)	
Female	320 (35.09)	34 (14.91)	41 (17.98)	103 (45.18)	142 (62.28)	
**Age, (years)**	51.13 ± 9.57	49.93 ± 9.45	51.56 ± 9.30	52.18 ± 9.18	50.85 ± 10.24	0.073
**BMI, (kg/m2)**	23.13 ± 3.12	26.48 ± 3.02	23.84 ± 1.66	22.19 ± 1.50	20.00 ± 1.60	***< 0.001***
**SBP, (mmHg)**	120.25 ± 14.96	126.74 ± 13.80	124.05 ± 13.40	118.18 ± 15.30	112.01 ± 12.85	***< 0.001***
**DBP, (mmHg)**	76.14 ± 10.01	80.95 ± 8.67	78.86 ± 9.03	74.57 ± 9.92	70.18 ± 8.87	***< 0.001***
**AST, (IU/L)**	20.85 ± 8.09	24.40 ± 11.28	20.54 ± 7.01	19.19 ± 5.66	19.28 ± 6.01	***< 0.001***
**ALT, (IU/L)**	22.69 ± 14.29	32.55 ± 20.94	23.57 ± 11.37	18.03 ± 7.00	16.60 ± 6.74	***< 0.001***
**GGT, (IU/L)**	19.00 (14.00, 28.00)	25.00 (19.00, 41.00)	21.00 (15.75, 32.25)	16.00 (13.00, 22.25)	14.00 (11.00, 18.00)	***< 0.001***
**FPG, (mg/dl)**	98.05 ± 14.06	103.43 ± 13.07	100.09 ± 19.84	95.94 ± 9.93	92.74 ± 8.03	***< 0.001***
**UA, (mg/dl)**	5.26 ± 1.38	6.09 ± 1.28	5.56 ± 1.24	4.90 ± 1.23	4.47 ± 1.18	***< 0.001***
**TC, (mg/dl)**	209.82 ± 35.97	219.71 ± 38.25	210.38 ± 32.80	204.79 ± 33.71	204.42 ± 36.92	***< 0.001***
**TG, (mg/dl)**	81.00 (53.00, 124.00)	155.00 (112.00, 202.75)	94.50 (75.00, 124.00)	65.00 (52.00, 81.25)	44.50 (34.00, 58.00)	***< 0.001***
**HDL-c, (mg/dl)**	53.54 ± 14.60	42.53 ± 9.85	49.31 ± 10.56	56.29 ± 12.08	66.01 ± 14.05	***< 0.001***
**LDL-c, (mg/dl)**	128.06 ± 31.69	136.40 ± 33.26	133.26 ± 29.67	125.49 ± 28.57	117.09 ± 31.65	***< 0.001***
**eGFR, (mL/min/1.73 m2)**	70.41 ± 12.04	68.47 ± 11.65	68.33 ± 10.28	70.47 ± 12.15	74.39 ± 13.00	***< 0.001***
ABI	1.19 (1.14, 1.24)	1.21 (1.15, 1.26)	1.20 (1.16, 1.24)	1.19 (1.14, 1.23)	1.16 (1.12, 1.20)	***< 0.001***
**Alcohol consumption, n (%)**						***0.003***
None or minimal	595 (65.24)	140 (61.4)	133 (58.33)	153 (67.11)	169 (74.12)	
Light	149 (16.34)	36 (15.79)	43 (18.86)	35 (15.35)	35 (15.35)	
Moderate	88 (9.65)	25 (10.96)	25 (10.96)	28 (12.28)	10 (4.39)	
Heavy	80 (8.77)	27 (11.84)	27 (11.84)	12 (5.26)	14 (6.14)	
**Smoking status, n (%)**						***< 0.001***
None or Past	715 (78.40)	160 (70.18)	177 (77.63)	179 (78.51)	199 (87.28)	
Current	197 (21.60)	68 (29.82)	51 (22.37)	49 (21.49)	29 (12.72)	
**Habit of exercise, n (%)**						***0.002***
No	719 (80.25)	200 (88.11)	179 (80.27)	175 (78.83)	165 (73.66)	
Yes	177 (19.75)	27 (11.89)	44 (19.73)	47 (21.17)	59 (26.34)	
**Fatty liver, n (%)**						***< 0.001***
No	646 (70.91)	72 (31.58)	152 (66.67)	198 (87.22)	224 (98.25)	
Yes	265 (29.09)	156 (68.42)	76 (33.33)	29 (12.78)	4 (1.75)	

BaPWV, brachial-ankle pulse wave velocity; 95% CI, 95% confidence interval; SPISE, single-point insulin sensitivity estimator; BMI, body mass index; SBP, systolic blood pressure; DBP, diastolic blood pressure; AST, aspartate aminotransferase; ALT, alanine aminotransferase; GGT, γ-glutamyltranspeptidase; FPG, fasting plasma glucose; UA, uric.acid; TC, total cholesterol; TG, triglyceride; HDL-c, high‐density lipoprotein cholesterol; LDL-C, low-density lipoprotein cholesterol; eGFR, estimated glomerular filtration rate; ABI, ankle-brachial index.

Bold values indicate statistically significant differences or associations (P < 0.05).

Across increasing SPISE quartiles, participants tended to have lower levels of BMI, SBP, DBP, AST, ALT, GGT, FPG, UA, TC, TG, LDL-C, and ABI, whereas HDL-C and eGFR levels increased gradually across SPISE quartiles; all differences were statistically significant (all P < 0.001).

The four groups also showed significant differences in lifestyle characteristics and fatty liver status (all P < 0.05). Higher SPISE levels were associated with lower smoking rates, a higher proportion of regular exercise, lighter alcohol consumption, and a lower prevalence of fatty liver, with the prevalence of fatty liver decreasing markedly from 68.42% to 1.75%.

### 3.2 Association of SPISE with baPWV and dose–response relationship

Univariate regression analysis (**Table 2**) showed that SPISE was significantly and inversely associated with baPWV. For each 1-unit increase in SPISE, baPWV decreased by 17.41 cm/s on average (P < 0.001). Significant positive associations with baPWV were observed for fatty liver, SBP, DBP, age, GGT, ALT, AST, TG, FPG, UA and TC, while HDL-C and eGFR showed significant inverse associations (all P < 0.05).

**Table 2:** Results of univariate analysis of baPWV.

Variable	β (95%CI)	P value
SPISE	-17.41 (-24.55, -10.27)	***< 0.001***
Sex, n (%)	-49.44 (-82.84, -16.05)	***0.004***
Age, (years)	12.95 (11.5, 14.39)	***< 0.001***
BMI, (kg/m2)	4.95 (-0.17, 10.07)	0.058
SBP, (mmHg)	8.43 (7.51, 9.35)	***< 0.001***
DBP, (mmHg)	11.29 (9.87, 12.71)	***< 0.001***
AST, (IU/L)	3.40 (1.43, 5.37)	***< 0.001***
ALT, (IU/L)	1.50 (0.38, 2.61)	***0.009***
GGT, (IU/L)	1.01 (0.36, 1.66)	***0.002***
FPG, (mg/dl)	4.21 (3.1, 5.31)	***< 0.001***
UA, (mg/dl)	22.77 (11.23, 34.31)	***< 0.001***
TC, (mg/dl)	0.71 (0.27, 1.16)	***0.002***
TG, (mg/dl)	0.44 (0.23, 0.65)	***< 0.001***
HDL-c, (mg/dl)	-1.33 (-2.42, -0.23)	***0.018***
LDL, (mg/dl)	0.66 (0.16, 1.17)	***0.010***
eGFR, (mL/min/1.73 m2)	-6.39 (-7.65, -5.12)	***< 0.001***
ABI	0.04 (-0.02, 0.1)	0.190
Habit of exercise, n (%)	16.65 (-23.27, 56.57)	0.413
Fatty liver, n (%)	93.74 (58.98, 128.51)	***< 0.001***
Alcohol consumption	0.07 (-0.06, 0.19)	0.284
Smoking status	-0.16 (-39.07, 38.75)	0.994

BaPWV, brachial-ankle pulse wave velocity; 95% CI, 95% confidence interval; SPISE, single-point insulin sensitivity estimator; BMI, body mass index; SBP, systolic blood pressure; DBP, diastolic blood pressure; AST, aspartate aminotransferase; ALT, alanine aminotransferase; GGT, γ-glutamyltranspeptidase; FPG, fasting plasma glucose; UA, uric acid; TC, total cholesterol; TG, triglyceride; HDL-c, high‐density lipoprotein cholesterol; LDL-C, low-density lipoprotein cholesterol; eGFR, estimated glomerular filtration rate; ABI, ankle-brachial index.

Bold values indicate statistically significant differences or associations (P < 0.05).

**Table 3** shows that higher SPISE was consistently associated with lower baPWV after adjustment for multiple covariates, whether SPISE was examined as a continuous variable or grouped by quartiles. When analyzed as a continuous variable, SPISE remained significantly negatively associated with baPWV in the fully adjusted model (β of -8.48, 95% CI: -16.00 to -0.96, P of 0.027). When analyzed by quartiles with Q1 as the reference group, participants in Q4 had significantly lower baPWV levels than those in Q1 (β of -56.17, 95% CI: -101.49 to -10.85, P of 0.015), and the trend test was statistically significant (P for trend of 0.013).

**Table 3:** Multivariable-adjust β and 95%CI of the SPISE index associated with baPWV.

Variable	Unadjusted		Model 1		Model 2		Model 3	
	β (95%CI)	P value	β (95%CI)	P value	β (95%CI)	P value	β (95%CI)	P value
SPISE	-17.41 (-24.54~-10.28)	<0.001	-15.38 (-22.07~-8.69)	<0.001	-8.75 (-16.22~-1.27)	0.022	-8.48 (-16~-0.96)	0.027
1st Quartile(≤6.46)	Ref		Ref		Ref		Ref	
2st Quartile (6.46-7.84)	-4.08 (-48.7~40.55)	0.858	-24.28 (-62.52~13.96)	0.214	-6.55 (-46~32.9)	0.745	-6.07 (-46.06~33.91)	0.766
3st Quartile (7.84-9.54)	-35.72 (-80.35~8.91)	0.117	-51.9 (-91.26~-12.53)	0.010	-22 (-64.41~20.42)	0.310	-21.18 (-63.79~21.42)	0.330
4st Quartile (≥9.54)	-104.01 (-148.63~-59.38)	<0.001	-94.87 (-135.76~-53.98)	<0.001	-57.32 (-102.37~-12.26)	0.013	-56.17 (-101.49~-10.85)	0.015
P for trend	-34.37 (-48.49~-20.24)	<0.001	-31.12 (-44.26~-17.99)	<0.001	-18.94 (-33.52~-4.36)	0.011	-18.58 (-33.23~-3.93)	0.013

Model 1 adjust for age, sex.

Model 2 adjust for Model 1+ ALT, AST, GGT, UA, eGFR, ABI.

Model 3 adjust for Model 1+ Model 2 + alcohol consumption, smoking status.

SPISE, single-point insulin sensitivity estimator; baPWV, brachial-ankle pulse wave velocity; 95% CI, 95% confidence interval; Ref, reference; BMI, body mass index; ALT, alanine aminotransferase; AST, aspartate aminotransferase; UA, uric acid; GGT, gamma-glutamyltransferase; eGFR, estimated glomerular filtration rate; ABI, ankle-brachial index.

**Figure 1:**
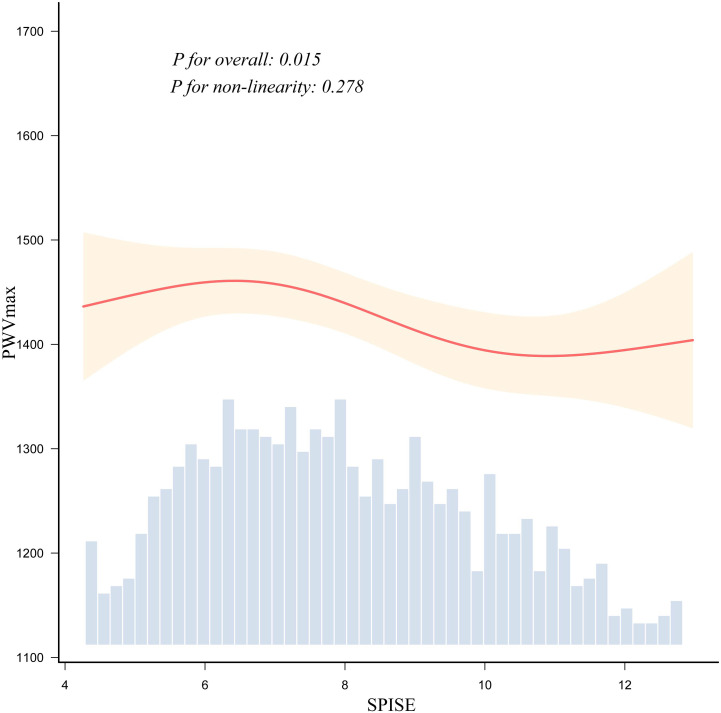
Restricted cubic spline analysis of the association between SPISE and baPWV. The solid red line represents the estimated association, and the shaded area indicates the 95% confidence interval. The histogram at the bottom shows the distribution of SPISE values in the study population. This RCS analysis was performed based on Model 3, with adjustment for age, sex, liver enzymes, uric acid, eGFR, ABI, alcohol consumption, and smoking status. To reduce the potential influence of extreme values, participants with SPISE values outside the mean ± 3 SD range were excluded from this analysis. The overall association between SPISE and baPWV was statistically significant (*P* for overall = 0.015), whereas no significant evidence of nonlinearity was observed (*P* for non-linearity = 0.278).

Quartile-based analyses showed a generally monotonic inverse association, with lower baPWV values observed at higher SPISE levels. After excluding participants with SPISE values outside the mean ± 3 SD range, the RCS analysis showed a significant overall association between SPISE and baPWV (*P* for overall = 0.015), with no significant evidence of nonlinearity (*P* for nonlinearity = 0.278) (**Figure 1**). The curve generally supported an inverse association across the main distribution range of SPISE.

To further evaluate the robustness of our findings, additional analyses were performed with further adjustment for cardiometabolic variables. After additional adjustment for TC and LDL-C (**Supplementary Table 1**), the inverse association between SPISE and baPWV remained largely unchanged, indicating that the results were robust to additional lipid adjustment. In contrast, further adjustment for SBP, DBP, and fasting plasma glucose (**Supplementary Table 2**) attenuated the association and rendered its direction less consistent, suggesting that blood pressure and related metabolic factors, particularly blood pressure, may play an important role in the SPISE–baPWV relationship.

### 3.3 Subgroup analyses of the association between SPISE and baPWV

**Figure 2:**
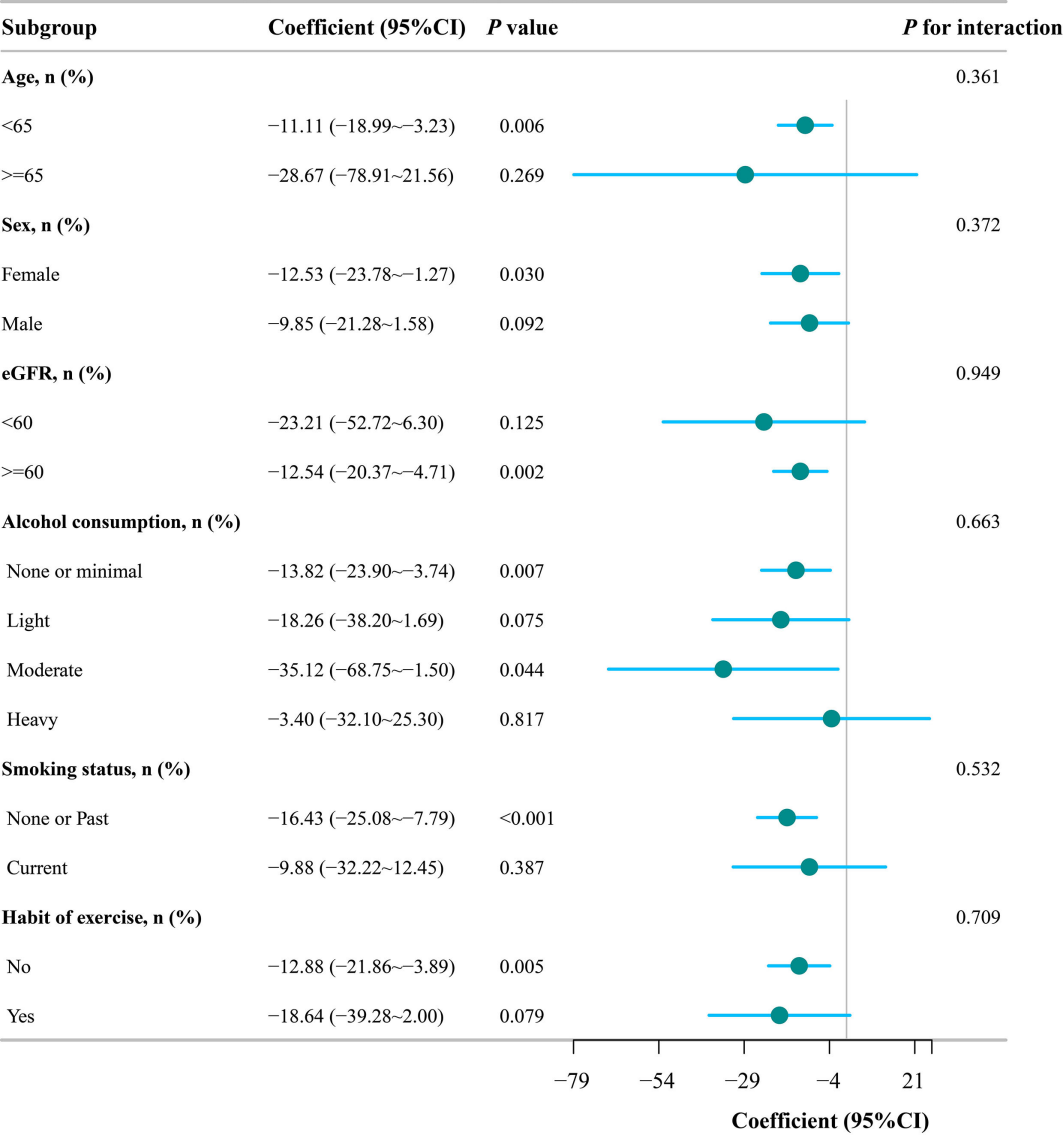
Subgroup analyses of the association between SPISE and baPWV. Dots indicate regression coefficients, and horizontal lines represent 95% confidence intervals. The vertical reference line indicates the null value. P values for interaction were calculated to assess potential effect modification across subgroups. No significant interaction was observed for age, sex, eGFR, alcohol consumption, smoking status, or exercise habits.

**Figure 2** presents the subgroup analysis. In general, the negative association between SPISE and baPWV was similar across the examined population strata. No significant interactions were observed for age, sex, eGFR, alcohol consumption, smoking status, or exercise habits (all P for interaction > 0.05), indicating no statistically significant effect modification. Although the association remained statistically significant in several strata, these subgroup-specific findings should be interpreted cautiously in the absence of significant interaction.

## 4 Discussion

In this cross-sectional analysis of 912 Japanese adults undergoing health check-ups, higher SPISE levels were associated with lower baPWV after multivariable adjustment. This inverse association remained evident after multivariable adjustment and was not accompanied by clear evidence of nonlinearity. In addition, the direction of association was generally consistent across subgroup analyses, although no statistically significant interaction was detected. Taken together, these findings suggest that SPISE may serve as an accessible metabolic marker associated with arterial stiffness in a health screening population.

Existing evidence has mainly concentrated on conventional insulin resistance indices, particularly HOMA-IR, in relation to arterial stiffness (7, 23). By contrast, data regarding SPISE, a more simplified and recently introduced index, in relation to baPWV are still scarce. Unlike SPISE, HOMA-IR depends on concurrent assessment of fasting glucose and insulin, which increases cost and may be affected by inter-assay variability in insulin measurement, thereby reducing its feasibility in large population studies (24). SPISE requires only three routine parameters—TG, HDL-C, and BMI—making it easy to calculate, and its correlation with insulin resistance has been validated in adolescent populations (25). Recent prospective cohort studies further indicate that higher SPISE levels are significantly associated with lower cardiovascular disease risk in middle-aged and older adults (26), but direct evidence linking SPISE to arterial stiffness in adult populations is still lacking. Against this background, the present study extends the existing literature by showing that SPISE is inversely associated with baPWV in a Japanese health check-up population, even after adjustment for multiple clinical and lifestyle-related factors.

The observed inverse association is biologically plausible. SPISE is essentially a positive indicator of insulin sensitivity, and insulin resistance is a major driver of increased arterial stiffness (6, 27). Impaired insulin signaling can reduce nitric oxide bioavailability, disrupt endothelial function, and promote vascular remodeling (28, 29). In parallel, chronic low-grade inflammation and metabolic dysregulation may accelerate elastin degradation, collagen deposition, and arterial wall stiffening (30, 31). Because SPISE incorporates information from BMI, TG, and HDL-C, it may reflect insulin sensitivity-related metabolic status more comprehensively than any single component alone. In the present study, participants with higher SPISE values tended to have a more favorable cardiometabolic profile, which may partly underlie the lower baPWV levels observed in this group.

Another notable finding was that the association between SPISE and baPWV appeared to be predominantly linear. Although some prior studies have suggested nonlinear associations between insulin resistance-related markers and vascular phenotypes (32, 33), our spline analysis did not demonstrate statistically significant nonlinearity. One possible explanation is that this health check-up population may represent a relatively early or moderate stage of metabolic disturbance, in which the relationship between insulin sensitivity and arterial stiffness is more gradual than threshold-dependent. However, this interpretation remains exploratory and should be confirmed in other populations. At the same time, the dose-response relationship should not be interpreted as a strictly simple linear negative correlation across the entire SPISE range. Considering the distribution of SPISE values, especially the relatively limited observations at the extreme ranges, the shape of the spline curve at both ends should be interpreted cautiously.

In addition, in sensitivity analyses, additional adjustment for SBP, DBP, and fasting plasma glucose substantially attenuated the inverse association between SPISE and baPWV and changed its direction. This suggests that blood pressure may play a central role in the SPISE–arterial stiffness relationship. One possible explanation is that blood pressure may serve not only as a confounder but also as a potential intermediate factor linking insulin sensitivity to arterial stiffness (34). Therefore, the inverse association observed in the primary models may be partly mediated through blood pressure-related pathways. Overall, these findings highlight the important role of blood pressure in this association, which warrants further investigation in future studies.

From a clinical and epidemiological perspective, SPISE has several practical advantages. It is derived from routinely available anthropometric and lipid parameters and does not require insulin measurement, making it easy to obtain in both large-scale studies and routine practice (35). baPWV, meanwhile, is a well-established noninvasive marker of arterial stiffness and a predictor of cardiovascular risk (36). Although SPISE cannot replace direct vascular assessment, the present findings suggest that it may provide complementary information for identifying individuals with an unfavorable metabolic profile potentially linked to increased arterial stiffness.

In subgroup analyses, the inverse association appeared to remain significant in several strata; however, no significant interaction was detected. Therefore, the subgroup findings should not be overinterpreted as evidence of true heterogeneity. Rather, they suggest that the overall inverse relationship between SPISE and baPWV is relatively stable across different demographic and lifestyle categories within this dataset.

This study has several strengths. First, it addresses a relatively underexplored question regarding the relationship between SPISE and arterial stiffness assessed by baPWV. Second, the study employed multiple statistical methods, including continuous and categorical variable analyses, trend tests, restricted cubic splines, and subgroup analyses, providing a comprehensive characterization of the relationship between the two. Third, the study adjusted for various potential confounders, including liver function, kidney function, uric acid, ankle-brachial index, and lifestyle factors, yielding relatively stable results.

At the same time, this study has several limitations. First, the cross-sectional design precludes the determination of causality; it cannot be inferred that a decrease in SPISE leads to a decrease in baPWV, as reverse causality or residual confounding may exist. Second, baPWV is a surrogate marker of arterial stiffness; although it is closely related to cardiovascular outcomes, it is not the gold standard for directly measuring aortic stiffness (e.g., cfPWV), and caution is needed when extrapolating results to other arterial stiffness indicators. Third, SPISE was calculated based on a single baseline measurement of biochemical indices and BMI, without considering long-term changes or intra-individual variability, which may underestimate the true strength of the association. Fourth, although this study adjusted for multiple confounding factors, there may still be unmeasured confounders, such as dietary patterns, physical activity intensity, family history, medication use, and direct inflammatory markers. Fifth, this study is a secondary analysis with limited available covariates.

## 5 Conclusion

In this cross-sectional study of Japanese adults undergoing health check-ups, higher SPISE levels were independently associated with lower baPWV. This inverse association remained significant after multivariable adjustment and showed no clear evidence of nonlinearity. The subgroup analyses further suggested that the association was generally consistent across different population strata, without statistically significant interaction. These findings indicate that SPISE, an easily obtainable surrogate marker of insulin sensitivity, may be associated with arterial stiffness in a health screening population. Further prospective studies are needed to clarify the temporal relationship between SPISE and arterial stiffness and to determine whether SPISE provides additional value in vascular risk assessment.

## Data Availability

The original contributions presented in the study are included in the article/**Supplementary Material**. Further inquiries can be directed to the corresponding authors.
